# BCMA/CD47-directed universal CAR-T cells exhibit excellent antitumor activity in multiple myeloma

**DOI:** 10.1186/s12951-024-02512-6

**Published:** 2024-05-23

**Authors:** Qizhong Lu, Hexian Li, Zhiguo Wu, Zhixiong Zhu, Zongliang Zhang, Donghui Yang, Aiping Tong

**Affiliations:** 1 Department of Biotherapy, State Key Laboratory of Biotherapy and Cancer Center, Research Unit of Gene and Immunotherapy, Chinese Academy of Medical Sciences, Collaborative Innovation Center of Biotherapy, West China Hospital, Sichuan University, Chengdu, 610041 China; 2 College of Veterinary Medicine, Shaanxi Center of Stem Cells Engineering and Technology, Northwest A&F University, Yangling, 712100 China; 3 Frontiers Medical Center, Tianfu Jincheng Laboratory, Chengdu, 610212 China

**Keywords:** Universal chimeric antigen receptor, Immunotherapy, Nanobody, Multiple myeloma

## Abstract

**Background:**

BCMA-directed autologous chimeric antigen receptor T (CAR-T) cells have shown excellent clinical efficacy in relapsed or refractory multiple myeloma (RRMM), however, the current preparation process for autologous CAR-T cells is complicated and costly. Moreover, the upregulation of CD47 expression has been observed in multiple myeloma, and anti-CD47 antibodies have shown remarkable results in clinical trials. Therefore, we focus on the development of BCMA/CD47-directed universal CAR-T (UCAR-T) cells to improve these limitations.

**Methods:**

In this study, we employed phage display technology to screen nanobodies against BCMA and CD47 protein, and determined the characterization of nanobodies. Furthermore, we simultaneously disrupted the endogenous TRAC and B2M genes of T cells using CRISPR/Cas9 system to generate TCR and HLA double knock-out T cells, and developed BCMA/CD47-directed UCAR-T cells and detected the antitumor activity in vitro and in vivo.

**Results:**

We obtained fourteen and one specific nanobodies against BCMA and CD47 protein from the immunized VHH library, respectively. BCMA/CD47-directed UCAR-T cells exhibited superior CAR expression (89.13-98.03%), and effectively killing primary human MM cells and MM cell lines. BCMA/CD47-directed UCAR-T cells demonstrated excellent antitumor activity against MM and prolonged the survival of tumor-engrafted NCG mice in vivo.

**Conclusions:**

This work demonstrated that BCMA/CD47-directed UCAR-T cells exhibited potent antitumor activity against MM in vitro and in vivo, which provides a potential strategy for the development of a novel “off-the-shelf” cellular immunotherapies for the treatment of multiple myeloma.

**Graphic Abstract:**

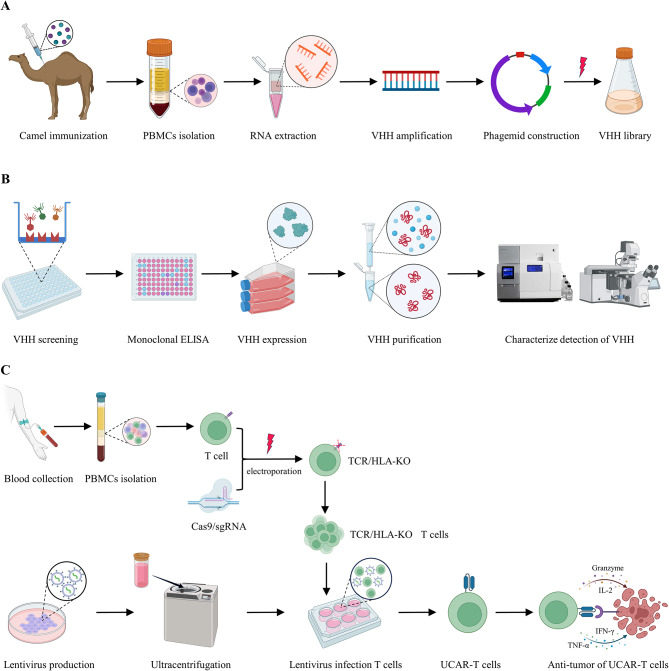

**Supplementary Information:**

The online version contains supplementary material available at 10.1186/s12951-024-02512-6.

## Introduction

Multiple myeloma (MM) is a neoplastic disease characterized by the abnormal proliferation of plasma cells (PCs) in the bone marrow and producing large amounts of pathological immunoglobulins, which accounts for 10% of hematological malignancies and has substantial mortality [1, 2]. The FDA has approved several immunotherapy agents for the treatment of MM, including monoclonal antibodies targeting CD38 (Daratumumab, Isatuximab) [3–5], CS1/SLAMF7 (Elotuzumab) [6], BCMA-directed antibody-drug conjugates (belantamab mafondotin) [7], BCMA-directed autologous CAR-T cells (Abecma, Carvykti) [8, 9], BCMA or GPRC5D-directed bispecific antibodies (Teclistamab, Elranatamab, Talquetamab) [10–12], proteasome inhibitors and immunomodulatory drugs [13, 14]. However, most MM patients experience relapse or become refractory due to the strong clonal heterogeneity of plasma cells, as well as target downregulation or loss [15–17]. As a result, MM remains an incurable disease, necessitating the development of new treatment approaches for these patients.

CAR-T cells have displayed promising efficacy in treating malignant cancers, especially for hematological malignancies [8, 9, 18, 19]. However, currently used CAR-T therapeutic agents are mainly derived from autologous T cells, which might be hampered by poor quality and quantity, laborious and costly. To overcome these limitations, autologous T cells may be replaced by allogeneic T cells from healthy donors, which through knock-out the endogenous TCR and B2M genes of T cells to avoid the occurrence of graft-versus-host disease (GvHD) [20–23].

BCMA (B-cell maturation antigen; CD269; TNFRSF17) is selectively over-expressed during the malignant transformation of plasma cells, making it an ideal target for the treatment of multiple myeloma [24]. It is a member of the tumor necrosis factor receptor superfamily and is expressed in normal and malignant plasma cells [25]. However, despite the overall response rates ranging from 63 to 100% in trials using BCMA-directed CAR-T cells [8, 26–28], approximately 45% of responders experience relapse due to BCMA downregulation or loss [29].

CD47 is a transmembrane glycoprotein that interacts with various proteins, including integrin, signal regulatory protein-α (SIRPα), thrombospondin (TSP)-1 and TSP-2 [30, 31]. The interaction between CD47 and SIRPα generates a “don’t eat me” signal to macrophages, inhibiting their phagocytic activity [32–34]. However, tumor cells have hijacked this mechanism by overexpressing CD47, allowing them to evade immune surveillance and leading to adverse clinical outcomes [35, 36]. To address, blocking the CD47-SIRPα interaction using antibodies has been shown to enhance antitumor immune responses [37–40]. Additionally, CD47-targeted CAR-T cells have effectively inhibited the growth of lung cancer, pancreatic cancer and ovarian cancer [41–43]. Recent studies have demonstrated the high expression of CD47 on malignancy PCs, further supporting CD47 as a potential immunotherapy target for multiple myeloma [39, 40, 44].

Nanobody (Nb) is a novel type of single-domain antibody fragment derived from camelids or sharks heavy-chain only antibodies (HcAbs), which have been widely used for developing novel biological agents due to their high affinity, small size (15 kDa), thermostability, and excellent tissue penetration characteristics [45–47]. Here, we obtained nanobodies against BCMA and CD47 protein from an immunized Bactrian camel by phage display technology, and constructed a novel BCMA/CD47-directed UCAR-T cells, which exhibit high safety for RBCs and effectively prolong the survival of mice compared to monospecific BCMA- or CD47-directed UCAR-T cells in xenograft models. This work provides a potential strategy for the development of a novel “off-the-shelf” cellular immunotherapies for the treatment of multiple myeloma.

## Materials and methods

### Cells and vectors

Human MM cell lines MM.1S, RPMI-8226, OPM-2, U266 and NCI-H929 were respectively purchased from ATCC and DSMZ, and they were cultured with RPMI-1640 medium (Gibco) supplemented with 10% fetal bovine serum (FBS, BI) at 37℃ in 5% CO_2_. All cell lines have been tested negative for contamination with mycoplasma. MM.1S-mCherry.ffLuc, RPMI-8226-mCherry.ffLuc, BCMA-GFP-Hela and CD47-GFP-Hela cells were prepared through lentivirus infection. pcDNA3.1 vector (cat# V79020, Invitrogen) was used for eukaryotic expression of the recombinant proteins, pMECS vector was used to construct phage library, LentiGuide-Puro (cat# 52963) and pSLCAR-CD19-BBz (cat# 135992) vectors were purchased from Addgene and used to prepare lentivirus.

### Gene cloning, protein expression, and purification of recombinant proteins

The gene sequence of BCMA-ECD (extracellular domain, 1-54 aa), CD47-ECD (extracellular domain, 19-139 aa) were respectively inserted into pcDNA3.1-MCS-mFc vector between *BamH* I and *Xho* I restriction sites [48]. Subsequently, the positive plasmids were transduced into HEK293T cells using polyetherimide reagent (PEI, Polysciences Inc) and cultured in 293™ Freestyle medium (Gibco). After 5 days of transduction, the culture supernatant containing target recombinant proteins was harvested and purified using Ni-NTA agarose (GE Healthcare). Finally, the expression and purity of the recombinant proteins were analyzed using SDS-PAGE.

### VHH library construction and screening of specific nanobodies

A healthy 4-year-old Bactrian camel was immunized with high-purity recombinant BCMA and CD47 extracellular domain protein through the subcutaneous route following previously reported procedures [49]. Peripheral blood lymphocytes (PBLs) were isolated from 200 mL fresh blood samples by density gradient separation. Total RNA was extracted from the PBMCs and reverse transcribed to cDNA using the SuperScript III First-Strand Synthesis System (cat# 18080051, Thermo Fisher). VHH genes were subsequently amplified using nested PCR, ligated into the pMECS vector and transformed into *E. coli* TG1 competent cells. The rate of VHH gene insertion was evaluated using PCR with MP57 and GIII primers [50].

To obtain the anti-BCMA and CD47 nanobodies, three consecutive rounds of bio-screening were performed. Briefly, purified CD22-mFc, BCMA-mFc and CD47-mFc protein were immobilized in microplate. For each round screening, CD22-mFc protein was used as negative control. The microplate wells were washed with PBS’T buffer (PBS with 0.05% Tween 20 (v/v)) and then were blocked with blocking buffer (PBS’T containing 5% (w/v) skimmed milk) at 37 °C for 1 h. Then, rescued recombinant phages were consecutive added to microplate wells that containing CD22-mFc, BCMA-mFc or CD47-mFc protein and incubated for 1 h at 37 °C, respectively. After washing, the retained phages were eluted with Glycine-HCl buffer (pH 3.0) and neutralized immediately with Tris-HCl buffer (pH 8.5) to a neutral condition. Next, the eluted phages were used to infect *E. coli* TG1 cells and amplified overnight at 37 °C after infecting with M13K07 helper phages. Subsequently, the amplified phages were purified using PEG 4000/NaCl precipitation for the next round bio-panning. After three rounds screening, anti-BCMA and anti-CD47 specific phage particles were enriched, and then 96 clones were picked randomly for monoclonal identification using an indirect ELISA with HRP-conjugated goat anti-M13 IgG antibody (cat# 11973-MM05T-H, SinoBiological). Finally, all positive clones (*P*/*N* ≥ 3.0, P: OD_450_ values of BCMA-mFc or CD47-mFc, N: OD_450_ values of CD22-mFc) were sequenced and grouped based on their complementary determining regions (CDRs) amino acid sequence.

### Identification the binding activity of Nbs

The activity of Nbs was determined by using indirect ELISA (iELISA), indirect immunofluorescent assay (IFA), and flow cytometry (FCM). For iELISA, BCMA-mFc or CD47-mFc recombinant proteins were coated overnight at 4℃ in the 96-well Immunoplates, washed, blocked with 5% skimmed milk, and then incubated with a dilution series of humanized nanobodies-hFc (huNbs-hFc) for 1 h at 37℃, and HRP-conjugated goat anti-human IgG (cat# 109-035-088, Jackson) was added and incubated for another hour. After washing, 3,3’,5,5’-tetramethylbenzidine (TMB) soluble reagent (cat# PR1200, Solarbio) was added and then the reaction was stopped with 2M H_2_SO_4_. The absorbance at 450 nm was measured using an automatic ELISA plate reader. Finally, the binding activity was evaluated using a four-parameter nonlinear regression curve fit (Graphpad Prism 9.0).

For IFA and FCM identification, the expression levels of BCMA on human MM cell lines were detected using APC anti-human BCMA antibody (cat# 357506, Biolegend). Additionally, Alexa Fluor® 594 rabbit anti-human IgG (cat# 309-585-003, Jackson) or APC anti-human IgG Fc antibody (cat# 410712, Biolegend) were incubated with MM cell lines. The z-stack images were acquired using an Olympus SpinSR10 instrument with 100 × objective lenses and analyzed using Olympus OlyVIA analysis software.

### Specificity identification of nanobodies

To identify the specificity of candidate Nbs against BCMA, recombinant proteins BCMA-mFc and CD22-mFc, CD123-mFc, CD276-mFc and mFc (2 µg/mL) were coated in the Immunoplates, respectively. Anti-BCMA Nbs and positive control antibody (11D5.3) [51] were incubated respectively with the above proteins, and HRP-conjugated goat anti-human IgG antibody was used to detect the binding activity.

### Humanization of nanobodies

Antibody humanization is important for reducing the immunogenic responses associated with animal-derived antibodies. Numerous antibodies are commercialized as therapeutics, however, the immunogenicity of animal-derived antibodies may restrict their application in clinical. According to previous research [52], the mutations of VHH conserved residues are Gln-1 ˃ Glu, Gln-5 > Glu, Glu-49 > Gly, Lys-95 > Arg, Pro/Thr-96 > Ala, and Gln-123 > Leu (numbers referred to the IMGT amino acid numbering (imgt.scientific chart)).

### SPR analysis

Surface plasmon resonance (SPR) was used to measure the affinity between antigen and antibody. The SPR-based measurements were conducted using Biacore 8 K from GE Healthcare. Firstly, anti-mouse antibodies were immobilized onto the flow cell of the CM5 sensor chip following the manufacturer’s guidelines. Then, BCMA-mFc or CD47-mFc protein (5 µg/mL) was captured at approximately 100 response units (RUs). For kinetic analysis, a twofold dilution series of antibodies were run across the chip, with a reference channel serving as the control. The obtained data was double-reference subtracted and fitted to a 1:1 binding model utilizing the Biacore Evaluation Software. Finally, the binding curves were represented graphically using Origin 9.0 software.

### RBCs binding and agglutination assay

To evaluate whether anti-CD47 nanobodies could bind to human red blood cells (RBCs) and cause agglutination, fresh human blood samples were collected from healthy donors. RBCs were obtained from the whole blood by centrifugation and resuspended with PBS to create a 2% (v/v) suspension. For RBCs binding activity assay, RBCs were incubated with a series concentration of humanized nanobodies, CC2C6 (cat# 323102, Biolegend) as the positive control antibody. Alexa Fluor™ 647 conjugated anti-human IgG Fc antibody (cat# A55749, Invitrogen) was used as a secondary antibody and the signals were quantified by FACS. For RBCs aggregation assay, the RBCs were mixed with an equal volume of antibodies serially diluted in a round-bottom 96-well plate, with CC2C6 as the control. RBC aggregation was observed after incubation at room temperature for 40 min.

### Detection the binding ability of humanized nanobodies with primary MM cells from multiple myeloma patients

Primary MM cells were obtained from newly diagnosed, relapsed, or post-treatment patients with multiple myeloma. Clinical samples were collected from the West China Hospital of Sichuan University following the hospital protocols. The primary MM cells were isolated and enriched by human CD138 Microbeads (cat# 130-051-301, Miltenyi Biotec) according to manufacture instructions. After blocking with human Fc blocker (cat# 564220, BD), hu388-hFc, hu404-hFc and h32-hFc fusions (50 nM) were incubated respectively with primary MM cells, and then stained with APC anti-human IgG Fc antibody (cat# 410711, Biolegend) and detected by flow cytometry.

### T cells isolation and culture

Peripheral blood mononuclear cells (PBMCs) were collected from healthy voluntary donors and isolated from the whole blood via Ficoll-Paque PLUS density gradient centrifugation (cat# 17144002, cytiva). CD3^+^ T cells were isolated using the Pan T cell isolation kit (cat# 130-096-535, Miltenyi Biotec). Next, the isolated CD3^+^ T cells were stimulated for 48 h with the human T cell activation/expansion kit (cat# 130-091-441, Miltenyi Biotec) in accordance with the manufacturer’s instructions. For this process, the T cells were cultured in X-VIVO™ 15 media (cat# 02-060Q, Lonza) supplemented with 10% inactivated FBS, 10 ng/mL human IL-7 (cat# 200-07, Peprotech), and 10 ng/mL human IL-15 (cat# 200-15, Peprotech) at a final concentration of 1 × 10^6^ cells/mL.

### CRISPR/Cas9-mediated gene knock-out

The sgRNAs targeting T cell receptor alpha constant (TRAC) and β2 microglobulin (B2M) were designed by CHOPCHOP (https://chopchop.cbu.uib.no), which respectively targeted the constant region to exon 1 of TCR α/β chain, and the functional region to exon 1 of B2M (Supplementary Table 1). To generate ribonucleoprotein complexes (RNPs), sgRNAs (5 µg each) and 10 µg recombinant Cas9 protein were mixed and incubated with 10 µL 4D-Nucleofector™ Solution at 37℃ for 15 min. Subsequently, activated T cells were completely mixed with RNPs and co-electroporated via the program E0-115 on Nucleofector 4D Device [21]. The knock-out efficiency of TRAC and B2M were estimated respectively using FITC anti-human β2-microglobulin antibody (cat# 395706, BioLegend), and PE anti-human TCR α/β antibody (cat# 306708, BioLegend).

### Genomic analysis of knock-out efficiency

To determine whether the TRAC and B2M genes of T cells were successfully knocked-out, genomic DNA was extracted from electroporated and un-electroporated control T cells using DNA purification kit (cat# A1120, Promega) following the manufacturer’s instructions. The amplified surrounding regions of the target sites were then confirmed by Sanger sequencing.

### Enrichment of gene-disrupted T cells

TCR and HLA-disrupted T cells were isolated using magnetic beads sorting as described in a previous study [21]. Briefly, T cells after electroporation 3-4 days were labelled consecutively with Biotin conjugated anti-human HLA-DR antibody (cat# 307614, Biolegend) and anti-human β2-microglobulin antibody (cat# 316308, Biolegend) according to the manufacturer’s instructions. Subsequently, gene-disrupted T cells were purified using anti-biotin microbeads (cat# 130-090-485, Miltenyi Biotec).

### CAR construction

The candidate huNb was used to construct CAR expression vectors based on the results of SPR. In brief, the sequence of anti-BCMA nanobody hu388 and anti-CD47 nanobody hu404 was cloned into the pSLCAR-CD19-BBz vector (cat# 135992, Addgene). The constructed vectors, CAR-h31 and CAR-h32 included the anti-BCMA nanobody hu388 and anti-CD47 nanobody hu404 were connected with (G_4_S)_3_ or (EAAAK)_3_ linker, respectively. In addition, the antibody 11D5.3 against BCMA, and Nb5 against CD123 protein as the positive and negative control, respectively.

### Lentiviral transduction of T cells

The CAR lentivirus production system was developed as previously reported [20]. Briefly, CAR-expressing plasmids, gag/pol expressing plasmid psPAX2, and VSV-G expressing plasmid pMD2.G were co-transfected into HEK293T cells. After 8 h of transfection, the medium was replaced with fresh DMEM supplemented with 10% FBS. The supernatant was harvested at 48 h and 72 h post-transduction, and then filtered through 0.45 μm-pore cellulose acetate membranes. Finally, the gene-disrupted T cells were infected with lentivirus at 32℃, 1000 × g for 2 h, and cultured at a final concentration of 1 × 10^6^ cells/mL in the presence of 10 ng/mL human IL-7 and IL-15.

### Detection the expression of CAR on gene-disrupted T cells

After 96 h of infection with CAR lentiviral, the T cells were analyzed for CAR expression using flow cytometry and immunofluorescence assay. The UCAR-T cells were blocked with human Fc blocker (cat# 564220, BD), followed by consecutive incubation with 5 µg/mL BCMA-mFc (CAR-hu388, CAR-h31 and CAR-h32) and CD47-hFc (CAR-hu404, CAR-h31 and CAR-h32) proteins, APC anti-human IgG Fc antibody (cat# 410711, Biolegend) and PE-goat anti-mouse IgG antibody (cat# 405307, Biolegend). PE-goat IgG isotype control antibody (cat# 403004, Biolegend) and APC Rat IgG2a, κ isotype control antibody (cat# 400511, Biolegend) was used as negative controls.

### In vitro functional assays

#### Real-time cell analysis (RTCA)

Cytotoxicity assays were measured using label-free RTCA instrument and E-Plate 96 (ACEA Biosciences) according to the manufacturer’s protocol. Initially, each well of the E-plate 96 was filled with 50 µL DMEM culture medium containing 10% FBS, and then BCMA/CD47-GFP-Hela cells (8 × 10^3^ cells/well) were seeded with 100 µL culture medium and cultured at 37℃ until the cell index (CI) value exceeded 1.0. Subsequently, the effector T cells (control or UCAR-engineered) were added at an effector-to-target (E:T) ratio of 2:1. The cell index-time plots were recorded by RTCA.

### Luciferase-based cytotoxicity assay and cytokine detection

MM.1S-mCherry.ffLuc and RPMI-8226-mCherry.ffLuc cells were co-cultured with UCAR-T cells at different E:T ratios (4:1, 2:1, 1:1, and 1:2) in RPMI-1640 medium for 24 h at 37℃. Subsequently, luminescence was measured using a Multi-Detection Microplate Reader (Tecan). In addition, primary MM cells were co-cultured respectively with UCAR-T cells for 24 h at 37℃. Moreover, the levels of IFN-γ, TNF-α, and IL-2 in the harvested supernatants were assessed according to the manufacturer’s instructions (IFN-γ cat# KHC4021, ThermoFisher; TNF-α cat# KAC1751, ThermoFisher; IL-2 cat# BMS221-2, ThermoFisher).

### Mouse xenograft studies

Xenograft mouse models of MM were established using NCG mice (NOD/ShiLtJGpt-Prkdc^em26Cd52^IL2rg^em26Cd22^/Gpt) aged 6 to 10 weeks. The mice received a tail vein injection of 2 × 10^6^ MM.1S-mCherry.ffLuc or RPMI-8226-mCherry.ffLuc tumor cells in 0.1 mL PBS. On day 7 after tumor delivery, the mice were randomly divided into five groups. Subsequently, a dose of 5 × 10^6^ UCAR-T cells in 0.1 mL sterile PBS was injected into the mice via the tail vein. Tumor growth was monitored using the IVIS Lumina In Vivo Imaging System (PerkinElmer). To visualize the tumors, the mice were injected with 150 mg/kg D-Luciferin potassium salt (cat# ST196, Beyotime) intraperitoneally and imaged with the IVIS. After 24 days of UCAR-T cells treatment, we collected peripheral blood of mice through the tail vein. The blood samples were incubated with BCMA-hFc (cat# 10620-H02H, SinoBiological), CD47-hFc or CD123-hFc (cat# 10518-H02H, SinoBiological) proteins, respectively, and stained with APC/Cy7 anti-human IgG Fc antibody (cat# 366912, Biolegend), and then analyzed by flow cytometry to determine the percentage of UCAR-T cells and mCherry cells.

### Statistical analysis

Statistical analyses were performed using Graphpad Prism version 9.0 software. Comparisons among multiple groups were performed using a two-way ANOVA test with a Bonferroni post-test. Statistical significance in Kaplan-Meier survival curves was assessed with the log-rank (Mantel-Cox) test. The difference was considered statistically significant when *P* < 0.05. *P < 0.05; **P < 0.01; ***P < 0.001; ****P < 0.0001; ns, no significance.

## Results

### BCMA and CD47 expression profiles in MM patients

**Fig. 1:**
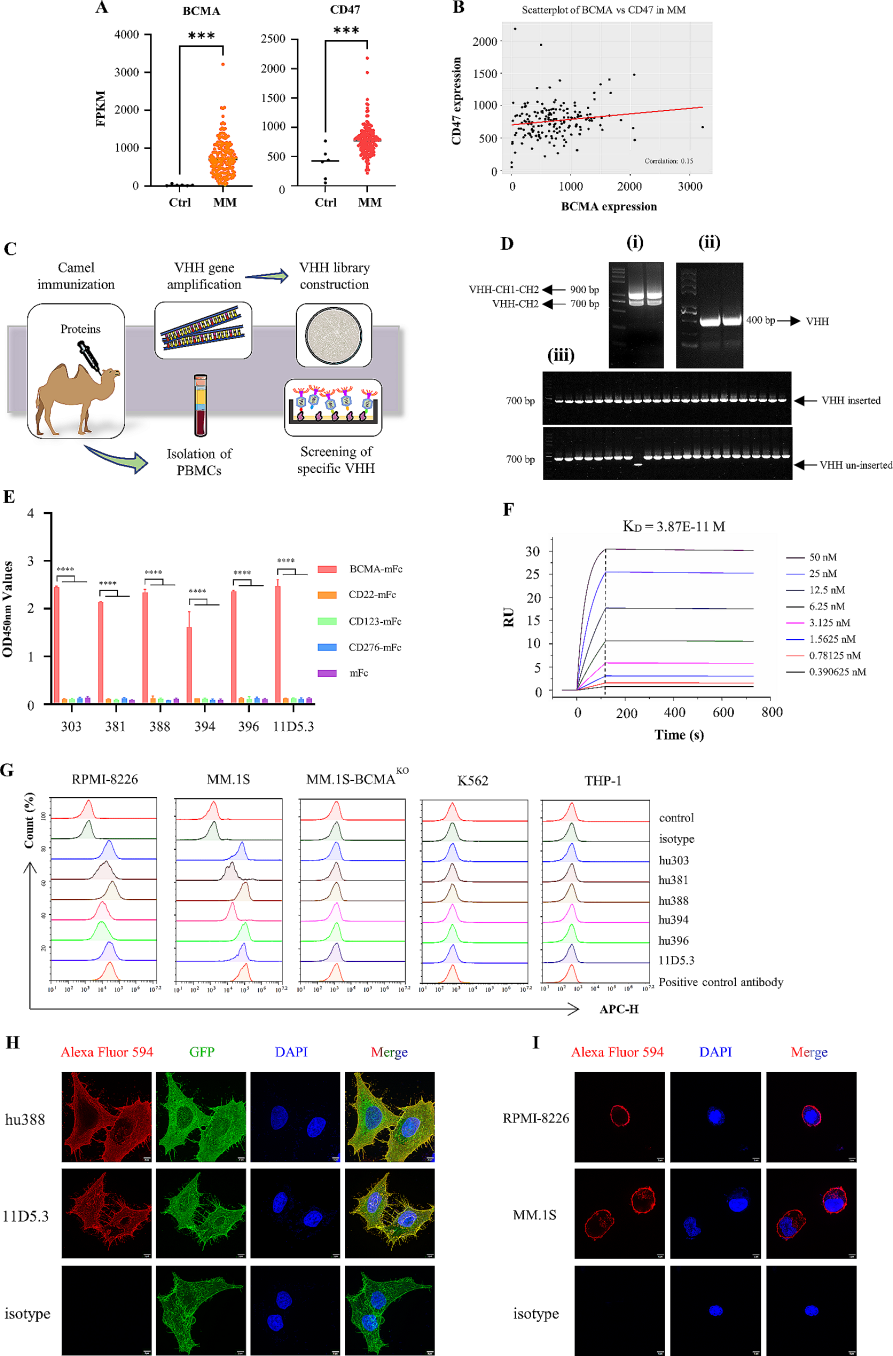
Screening specific nanobodies, and identification their functional characterization. (A) The transcript expression levels of BCMA and CD47 in MM patients (*n* = 170) was significantly higher than control (*n* = 6) (*P* < 0.001) from MMRF-CoMMpass data. (B) The correlation of expression between BCMA and CD47 is 0.15, indicating no significant correlation. (C) Schematic presentation of library construction and screening nanobodies by phage display technology. (D) Amplification of VHH gene (400 bp) by nest-PCR from PBMCs and estimation the VHH gene insert rate of nanobodies library. The VHH-CH1-CH2 (900 bp), VHH-CH2 (700 bp) gene of camel IgG were amplified after first round PCR (i), and VHH gene (400 bp) was amplified after second round PCR (ii). After amplification with MP57 and GIII primers, the PCR product containing the VHH gene is 700 bp, whereas the un-inserted product is 357 bp (iii). (E) The candidate nanobodies Nb303, Nb381, Nb388, Nb394 and Nb396 against BCMA could specifically binding to BCMA protein according to iELISA. (F) Real-time binding profile of anti-BCMA humanized nanobody hu388 with BCMA protein by SPR. The binding affinity between hu388 and BCMA protein was 3.87 × 10^− 11^M. (G) Specificity identification of anti-BCMA humanized nanobodies by flow cytometry. Humanized nanobodies hu303, hu381, hu388, hu394 and hu396 could specifically bind to RPMI-8226 and MM.1S cells, and not with MM.1S-BCMA^KO^, K562 and THP-1 cells. (H-I) Anti-BCMA humanized nanobody hu388 showed strong binding ability with BCMA protein on the BCMA-GFP-Hela cells (H), RPMI-8226 and MM.1S cells (I), 11D5.3 was positive control antibody against BCMA. Alexa Fluor® 594 rabbit anti-human IgG as the secondary antibody for detection. Scale bar 5 μm. Results were expressed as mean ± SD, statistical analyses were performed using *t*-test (A) and two-way ANOVA test with Dunnett’s multiple comparisons test (E). *P < 0.05, **P < 0.01, ***P < 0.001, **** *P* < 0.0001; ns, no significance

The mRNA expression levels of BCMA and CD47 in the MM patients was analyzed according to the Multiple Myeloma Research Foundation (MMRF) CoMMpass data (GSE accession: 39754, 170 newly diagnosed MM patients and 6 controls), and results revealed that the mRNA expression levels of BCMA and CD47 was significantly higher than control (*P* < 0.001) (Fig. 1A). Additionally, the correlation between BCMA and CD47 is 0.15, indicating no significant correlation (Fig. 1B), which supports the potential of a BCMA/CD47-targeting therapeutic strategy for multiple myeloma.

### Construction of VHH library and screening of specific nanobodies

The general process to obtain high-affinity specific nanobodies includes protein production, camel immunization, VHH library construction, and nanobodies screening (Fig. 1C). Firstly, high-purity BCMA-mFc (Fig. S1A) and CD47-mFc (Fig. S1B) recombinant proteins were obtained through HEK293T cells. After immunization, VHH-CH1-CH2 (900 bp) and VHH-CH1 (700 bp) genes from camel IgG were amplified after first round PCR with the RNA as the template that extracted from camel’s PBMCs, and then VHH (400 bp) was amplified after second round PCR with VHH-CH1 gene as the template (Fig. 1D). Following digestion, ligation and transformation, a phage display immunized VHH library was successfully constructed, containing approximately 2.77 × 10^9^ individual clones and the insert rate of VHH gene was approximately 97.91% (Fig. 1D). Specific nanobodies were screened using phage display technology. Three consecutive rounds of bio-screening were conducted to enrich specific nanobodies particles. From 90 BCMA and 94 CD47 binding clones (Fig. S1D), sequencing of the CDRs revealed that based on the amino acid sequences fourteen different BCMA-specific nanobodies and only one CD47-specific nanobody were obtained (data not shown).

### Nanobodies show excellent binding ability with BCMA protein

The expression and purification procedures of Nbs were conducted based on previous research [48]. High-purity anti-BCMA Nbs-hFc fusions (Fig. S2A) and anti-CD47 humanized nanobody hu404-hFc (Fig. S2B) were generated. iELISA results showed that anti-BCMA nanobodies could bind to BCMA protein (Fig. S2C). To further identify higher affinity nanobodies, a screening process was performed using 0.1 µg/mL nanobodies and a dilution series of BCMA protein (400, 200, 100, and 50 ng/well). Results showed that Nb303, Nb381, Nb388, Nb394, and Nb396 have higher affinity with BCMA protein compared to other anti-BCMA nanobodies (Fig. S2D). Additionally, these five candidate nanobodies and anti-BCMA positive control antibody 11D5.3 specifically bind to BCMA protein and not with CD22, CD123, CD276 and mFc proteins (Fig. 1E).

### Functional characterization of humanized nanobodies

SPR has been widely known as a golden standard for measuring antibodies affinity. The binding of nanobodies with BCMA protein was confirmed via SPR. SPR results revealed that the binding affinity of hu388 with BCMA protein was 3.87 × 10^− 11^ M (Fig. 1F), and other anti-BCMA nanobodies and 11D5.3 antibody binding ability from 10^− 11^ M to 10^− 9^ M (Fig. S2E). Moreover, iELISA results showed that humanized nanobodies hu388 (Fig. S2F) and hu404 (Fig. S2G) have better binding ability with BCMA or CD47 protein compared with original camelid nanobodies. Additionally, the BCMA gene of MM.1S cells was knocked-out using Cas9/sgRNA complex (Supplementary Table 1), sequencing data confirmed the successful knock-out of BCMA from MM.1S cell lines (Supplementary Table 2). Furthermore, flow cytometry results indicated that anti-BCMA humanized nanobodies could specifically bind to RPMI-8226 and MM.1S cells, and not with MM.1S-BCMA^KO^, K562 and THP-1 cells (Fig. 1G). Meanwhile, IFA results indicated that anti-BCMA antibodies hu388 and 11D5.3 strongly bind to BCMA-GFP-Hela cells (Fig. 1H), RPMI-8226 and MM.1S cells (Fig. 1I). Taken together, humanized nanobodies hu388 and hu404 were chosen as candidates for further development.

### Anti-CD47 nanobodies show high affinity with CD47 protein

**Fig. 2:**
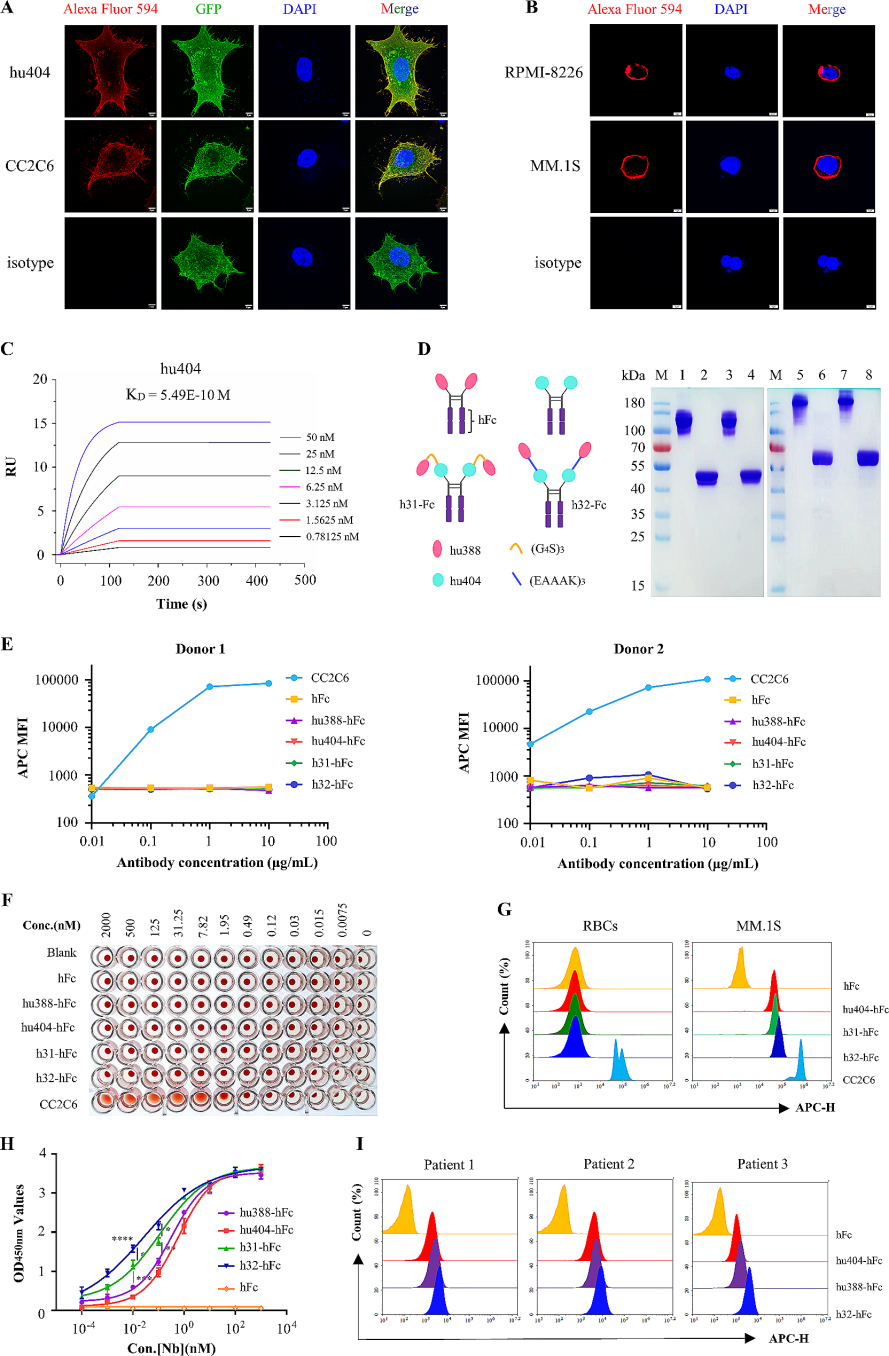
Anti-CD47 nanobodies hu404 has strong binding ability with CD47 protein, and showed high safety for human RBCs. (A-B) Immunofluorescence assay showed anti-CD47 nanobody hu404 has strong binding ability with CD47 protein on the CD47-GFP-Hela cells (A), RPMI-8226 and MM.1S cell lines (B). Scale bar 5 μm. (C) Real-time binding profile showed the binding affinity between hu404 and CD47 was 5.49 × 10^− 10^ M. (D) The tandem linked of hu388 and hu404 with flexibility linker (3×G_4_S) or rigidity linker (3×EAAAK) were obtained after purification with Ni-NTA agarose. M: Marker; lane 1,3,5,7 and lane 2,4,6,8: non-reducing and reducing of hu388-hFc, hu404-hFc, h31-hFc and h32-hFc. (E) Anti-CD47 nanobodies hu404-hFc, h31-hFc and h32-hFc fusions did not bind to human RBCs from healthy donors. (F) Anti-CD47 nanobodies hu404-hFc, h31-hFc and h32-hFc fusions did not agglutinate human RBCs. (G) Anti-CD47 nanobody hu404-hFc, h31-hFc and h32-hFc fusions did not bind to human RBCs, but still bind to MM.1S cells. (H) iELISA revealed the binding affinity of bispecific antibodies (h31-hFc, h32-hFc) were significantly higher than monospecific antibodies at 0.1 and 0.01 nM (*P* < 0.01), and h32-hFc shows the highest affinity (*P* < 0.0001). (I) The humanized nanobodies hu404-hfc, hu388-hFc and h32-hFc fusions showed strong binding activity with primary MM cells from multiple myeloma patients. Results were expressed as mean ± SD, statistical analyses were performed using a two-way ANOVA test with Bonferroni post-test. *P < 0.05, **P < 0.01, ***P < 0.001, **** *P* < 0.0001; ns, no significance

CD47-GFP-Hela, RPMI-8226 and MM.1S cell lines were stained respectively with anti-CD47 nanobody hu404-hFc fusion, anti-CD47 antibody (CC2C6, Biolegend) as the positive control antibody. The results demonstrated that anti-CD47 nanobody hu404-hFc fusion can strongly bind to CD47 protein on the surface of CD47-GFP-Hela cells (Fig. 2A), RPMI-8226 and MM.1S cells (Fig. 2B). Furthermore, SPR results demonstrated that anti-CD47 nanobody hu404 have high affinity with CD47 protein (K_D_ = 5.49E-10 M) (Fig. 2C).

### Anti-CD47 nanobodies hu404 show high safety for human RBCs

The bispecific antibodies, h31-hFc and h32-hFc, were generated by linking hu388 and hu404 via a flexible linker ((G_4_S)_3_) or a rigid linker ((EAAAK)_3_), as depicted in Fig. 2D. SDS-PAGE analysis confirmed the successful expression and purification of h31-hFc and h32-hFc fusions (Fig. 2D). To assess the potential adverse events of hu404, including anemia and thrombocytopenia, an RBCs binding and agglutination assay was performed in vitro. As predicted, the anti-CD47 antibody (CC2C6) exhibited a strong binding to RBCs. In contrast, the binding ability of hu404-hFc fusion to human erythrocytes was negligible (Fig. 2E) and did not cause human hemagglutination ranging from 2000 to 0.0075 nM (Fig. 2F). Furthermore, to determine whether recombinant nanobodies could maintain their tumor cell targeting ability in the presence of large RBCs. MM.1S cells were mixed with approximately 500-fold excess of human RBCs, and then incubated with recombinant nanobodies. Flow cytometry results revealed that anti-CD47 humanized nanobodies hu404-hFc fusion did not bind to human RBCs but still effectively bind to MM.1S cells (Fig. 2G).

### Bispecific antibodies show better binding ability than monospecific antibodies

To compare the binding ability between bispecific antibodies (h31-hFc, h32-hFc) and monospecific antibodies (hu388-hFc, hu404-hFc). Both BCMA-mFc and CD47-mFc protein (2 µg/mL) were co-coated in Immunoplates wells, and then hu388-hFc, hu404-hFc, h31-hFc, h32-hFc and hFc fusions (10^3^-10^− 4^ nM) were incubated with coated antigen, respectively. iELISA results revealed that bispecific antibodies (h31-hFc, h32-hFc) exhibited significantly higher binding affinity than monospecific antibodies at 0.1 and 0.01 nM (*P* < 0.01), and h32-hFc fusion exhibited the highest affinity (*P* < 0.0001) (Fig. 2H). Furthermore, primary human MM cells from multiple myeloma patients were isolated and enriched by CD138 Microbeads, and flow cytometry results revealed that humanized nanobodies hu404-hfc, hu388-hFc and h32-hFc fusions showed strong binding ability with primary MM cells, and h32-hFc fusion showed better binding activity than hu404-hFc and hu388-hFc fusions (Fig. 2I).

### Design and functional characterization of CAR

In this work, the structures of the CARs include a CD8 signal peptide, antigen-binding domain, CD8 ectodomain (EC), CD28 transmembrane domain (TM), 4-1BB intracellular costimulatory domain and a CD3ζ signal transduction domain. The mono- and bispecific anti-MM CARs scheme are shown in Fig. S4A. The structures of the CARs on the T cells are presented in Fig. S4B. For simplicity, bispecific CAR architectures are named CAR-h31 and CAR-h32, which contain flexible linker (“F” for 3 × G_4_S linkers) and rigid linker (“R” for 3 × EAAAK linker), respectively. Additionally, Fig. S4C outlines the general process used to produce UCAR-T cells with lentivirus, including T cells isolation and activation, TCR and HLA double knock-out, TCR^-^/HLA^-^-T cells enrichment, lentivirus transduction and expansion.

### Generation of gene-disrupted T cells

**Fig. 3:**
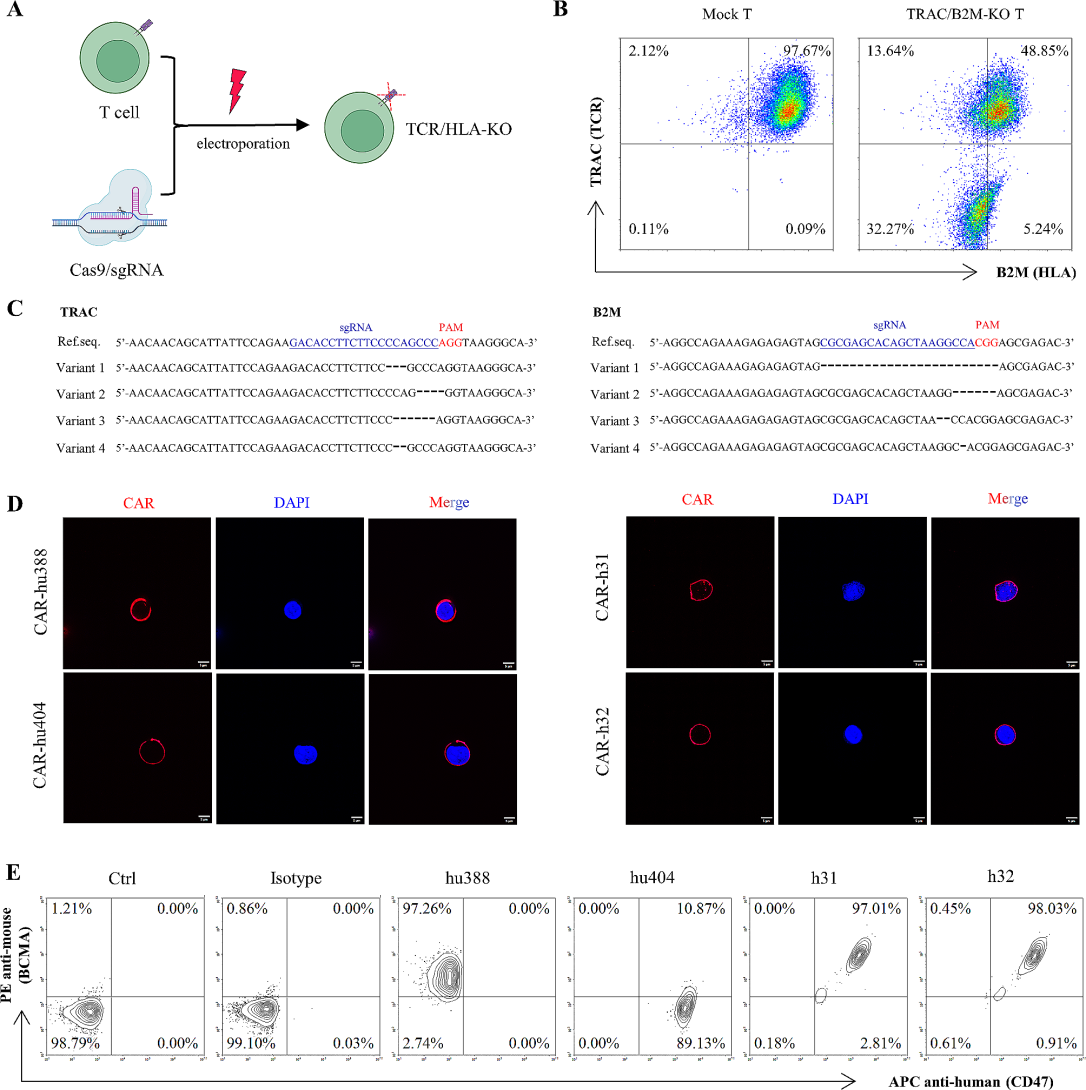
Generation of gene-disrupted T cells and identification the expression of CAR on the surface of human T cells. (A) Schematic presentation of gene-disrupted T cells generation, Cas9/sgRNA RNP complexes were electroporated into T cells. (B) The TCR and HLA double knockout rate of T cells was 32.27% after using Cas9/sgRNA RNP complexes electroporation. (C) Schematic presentation of mutated DNA sequences of *TRAC* (left) and *B2M* (right) in the TCR and HLA knock-out T cells. (D-E) Anti-MM CARs were efficiently expressed on the surface of human gene-disrupted T cells detected by immunofluorescence assay (D) and flow cytometry (E). Scale bar 5 μm

To disrupt TCR and HLA expression of T cells, TRAC and B2M-targeted sgRNA (Supplementary Table 1) and Cas9 protein was co-electroporated into T cells, and TCR and HLA were knocked-out (Fig. 3A). The flow cytometry staining of T cell surface TCR-KO and HLA-KO results showed that the TCR and HLA double-knockout rate of T cells was 32.27% (Fig. 3B). Furthermore, mutated DNA sequences confirmed the presence of on-target edits at *TRAC* (left) and *B2M* (right) in the TCR and HLA double knock-out T cells (Fig. 3C). Finally, TCR and HLA double knock-out T cells were purified using anti-biotin microbeads.

### Specific CARs are efficiently expressed on the surface of human T cells

After transduction 96 h with lentivirus, T cells were incubated with BCMA-mFc (Fig. S1A) and CD47-hFc proteins (Fig. S1C) to investigate the efficacy of CAR expression on the T cell surface. The results revealed that the successful expression of both monospecific and bispecific CARs on gene-disrupted T cells (Fig. 3D), with expression rates ranging from 89.13 to 98.03% after a single round transduction (Fig. 3E).

### Gene disruption did not alter CAR-T functions

**Fig. 4:**
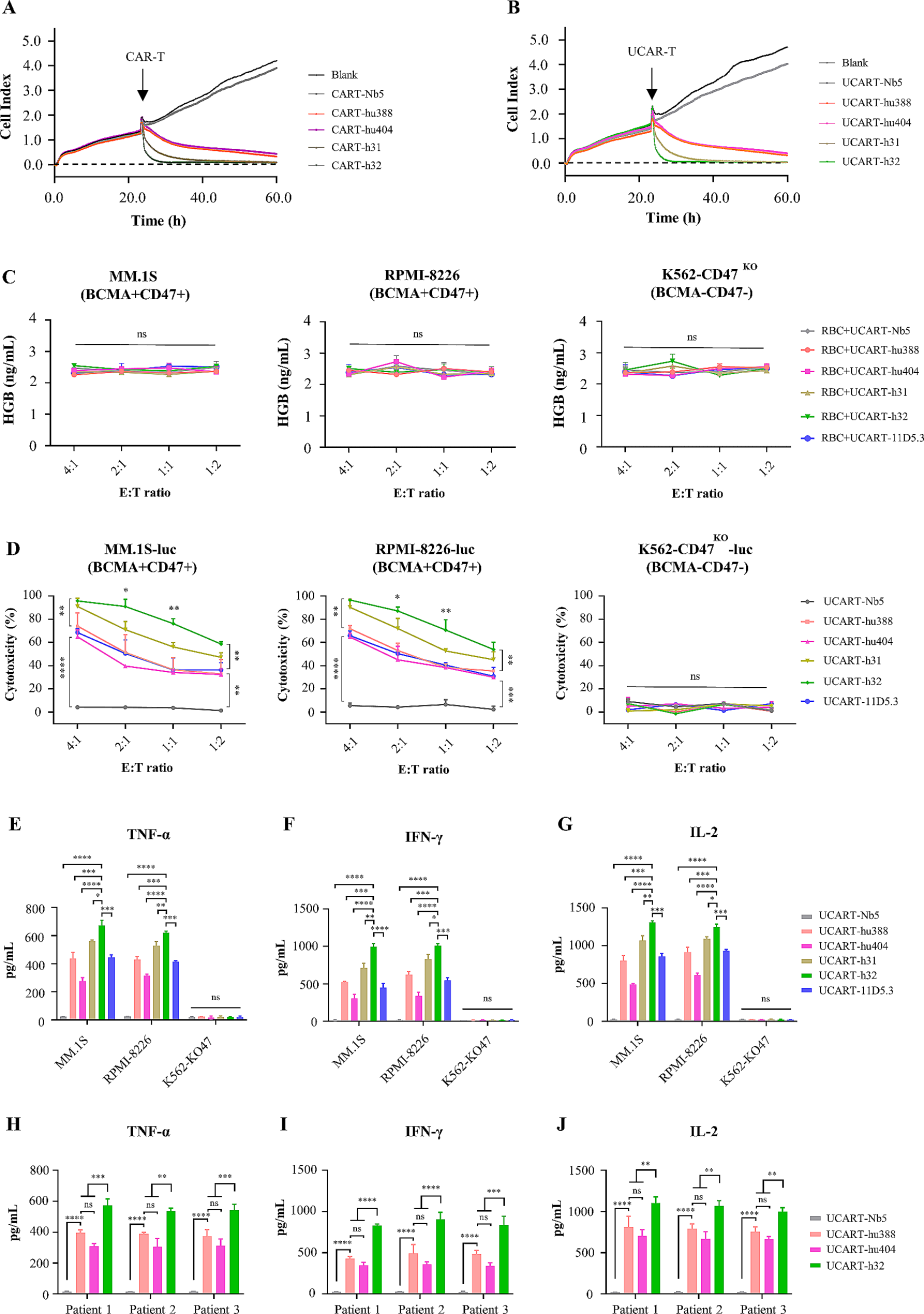
Cytotoxicity of anti-MM UCAR-T cells against MM targeting cells in vitro. (A-B) Comparison the anti-tumor activity between general CAR-T cells (A) and UCAR-T cells (B) via RTCA assay. Results showed that gene-disrupted did not alter CAR-T cells anti-tumor functions. (C) The HGB levels in the supernatant were no significant difference among the co-cultured of RBCs, tumor cells and UCAR-T cells in vitro. (D) BCMA/CD47-directed UCAR-T cells (h31-UCART, h32-UCART) showed strong specific cytolysis than monospecific UCAR-T cells (hu388-UCART, hu404-UCART) for human MM cell lines MM.1S and RPMI-8226. (E-G) The levels of TNF-α (E), IFN-γ (F) and IL-2 (G) treated with BCMA/CD47-directed UCAR-T cells were significantly higher than treated with monospecific UCAR-T cells for MM.1S and RPMI-8226 cell lines. (H-J) The levels of TNF-α (H), IFN-γ (I) and IL-2 (J) treated with BCMA/CD47-directed h32-UCART cells were significantly higher than treated with monospecific UCAR-T cells for primary MM cells. Results were expressed as mean ± SD, statistical analyses were performed by two-way ANOVA test followed by Tukey’s post-test analysis performed for all comparisons (C-D, H-J), a one-way ANOVA test with Tukey’s post-test analysis (E-G). *P < 0.05, **P < 0.01, ***P < 0.001, **** *P* < 0.0001; ns, no significance

To evaluate whether CRISPR/Cas9 gene-editing affect the antitumor function of T cells. We detected their responses to BCMA/CD47-GFP-Hela cells compared to un-electroporated control CAR-T cells using RTCA in vitro. The results showed that the antitumor efficacy of UCAR-T cells was equal to that of the wild-type CAR-T cells, and the effector function of CAR-h32 was higher than others (Fig. 4A-B). These findings revealed that the antitumor function of UCAR-T cells would not be affected by CRISPR/Cas9 editing of the endogenous TCR for adoptive immunotherapy.

### Bispecific UCAR-T cells exert strong cytotoxic effects against MM cells

To detect the safety of UCAR-T cells for RBCs, we co-cultured RBCs, BCMA^+^/CD47^+^ cells (MM.1S and RPMI-8226), BCMA^−^/CD47^−^ cells (K562-CD47^KO^) and UCAR-T cells with different E:T ratio. The results revealed no significant difference in the HGB levels between BCMA, CD47 and CD123-targeted UCAR-T cells in the MM.1S, RPMI-8226 and K562-CD47^KO^ cells (*P* > 0.05) (Fig. 4C), which suggest that the use of UCAR-T cells targeting CD47 is safe for RBCs. Comparative analysis revealed that the cytotoxicity of bispecific UCAR-T cells was stronger than monospecific UCAR-T cells (*P* < 0.01) (Fig. 4D). Furthermore, h32-UCART demonstrated superior activity compared to h31-UCART cells (*P* < 0.05), while there was no statistically significant difference in the kill of MM.1S and RPMI-8226 cells by hu388-UCART and 11D5.3-UCART cells (*P* > 0.05) (Fig. 4D). As shown in Fig. 4E-G, the levels of TNF-α, IFN-γ and IL-2 were higher in BCMA/CD47-directed UCAR-T cells compared to monospecific UCAR-T cells (*P* < 0.05). Specifically, the levels of culturing h32-UCART cells with target cells were higher than those of h31-UCART (*P* < 0.05), indicating that the antitumor activity of h32-UCART was strongest. Furthermore, hu388-UCART, hu404-UCART and h32-UCART cells were co-cultured with primary MM cells, and the levels of TNF-α, IFN-γ and IL-2 in h32-UCART cells were significantly high than hu388-UCART and hu404-UCART cells (*P* < 0.01) (Fig. 4H-J).

### Bispecific BCMA/CD47-directed UCAR-T outpace monospecific UCAR-T cells in vivo

**Fig. 5:**
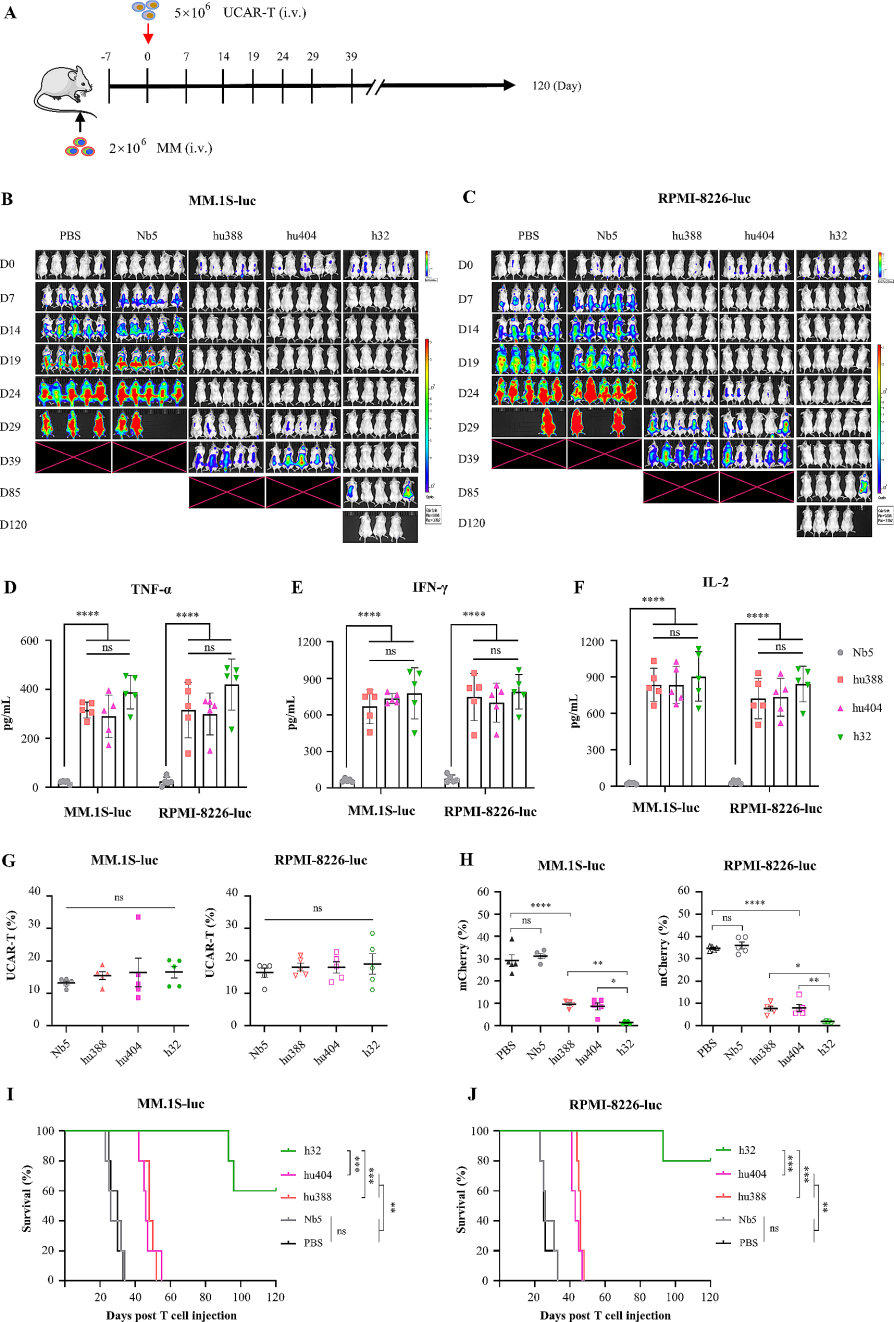
Cytotoxicity of anti-MM UCAR-T cells against MM targeting cells in NCG xenograft models. (A) Schematic of the tumor models in which 2 × 10^6^ tumor cells were injected (i.v.). Mice received 5 × 10^6^ UCAR-T cells at day 0 and monitor tumor burden, body weight and survival. (B-C) RPMI-8226-mCherry.ffLuc and MM.1S-mCherry.ffLuc cells were injected i.v. into NCG mice, and treated beginning on day 0 with PBS, Nb5-UCART against CD123 and anti-MM UCAR-T cells (*n* = 5). MM cells were effectively limited in mice treated with monospecific and bispecific UCAR-T cells compared to Nb5-UCART cells and PBS control. (D-F) The levels of TNF-α, IFN-γ and IL-2 in mice treated with anti-MM UCAR-T cells was significantly higher than treated with Nb5-UCART cells against CD123 (*n* = 5). (G) The proportion of UCAR-T cells in the peripheral blood (PB) of mice showed no significant difference on day 24 after UCAR-T cells treatment (*n* = 5). (H) The proportion of mCherry cells in the PB of mice on day 24 after monospecific UCAR-T cells treatment was higher than BCMA/CD47-directed h32-UCART cells (*n* = 5). (I-J) Kaplan-Meier analysis showed the mice treated with BCMA/CD47-directed h32-UCART cells had significantly longer survival compared to treated with monospecific UCAR-T cells (hu388-UCART and hu404-UCART). A log-rank Mantel-Cox test was used to test for statistical significance. Data represent mean ± SD, statistical significance was determined by a one-way ANOVA with Tukey’s post-test. *P < 0.05, **P < 0.01, ***P < 0.001, **** *P* < 0.0001; ns, no significance

To evaluate the antitumor activity of bispecific BCMA/CD47-directed UCAR-T cells in vivo, NCG mice were engrafted with MM.1S-mCherry.ffLuc and RPMI-8226-mCherry.ffLuc cells (i.v.) and then treated with UCAR-T cells (Fig. 5A). Bioluminescent imaging (BLI) revealed that MM cells were effectively limited in mice treated with BCMA or CD47-directed UCAR-T cells and BCMA/CD47-directed UCAR-T cells compared to CD123-directed Nb5-UCART cells and PBS control (Fig. 5B-C, Fig.S5A). However, mice treated with monospecific BCMA or CD47-directed UCAR-T cells (hu388-UCART and hu404-UCART) relapsed on day 29 and day 24 in MM.1S and RPMI-8226 model, respectively (Fig. 5B-C). After 10 days of UCAR-T cells treatment, the levels of TNF-α, IFN-γ and IL-2 in mice treated with anti-MM UCAR-T cells was significantly higher than Nb5-UCART cells (Fig. 5D-F). By analyzing the proportion of UCAR-T cells and mCherry cells in the peripheral blood of mice on day 24 after UCAR-T cells treatment, it was found that there was no significant difference in the proportion of UCAR-T cells (*P* > 0.05) (Fig. 5G). Furthermore, the proportion of mCherry cells was higher treated with monospecific UCAR-T cells compared to bispecific BCMA/CD47-directed UCAR-T cells (*P* < 0.05) (Fig. 5H). Kaplan-Meier endpoint survival curves in Fig. 5I-J demonstrated that the mice treated with BCMA/CD47-directed UCAR-T cells (h32-UCART) had significantly longer survival compared to treated with monospecific UCAR-T cells (hu388-UCART and hu404-UCART) (*P* < 0.001). Additionally, the body weights of mice treated with h32-UCART cells were increased (Fig. S5B), the body weights loss of mice treated with hu388-UCART or hu404-UCART cells less than 10% (Fig. S5B), and clinical signs of GvHD like hair loss, altered posture, or reduced mobility were not observed, which indicate there are no adverse immune reactivities.

## Discussion

The FDA has approved Abecma and Carvykti, BCMA-directed CAR-T cells for the treatment of patients with relapsed or refractory multiple myeloma (RRMM), which results in high remission rates (72-97% ORR) in patients with RRMM [8, 9]. However, downregulation or loss of BCMA expression has been observed in a few MM patients after BCMA-directed CAR-T cell therapy, which mediates multiple myeloma resistance to anti-BCMA CAR-T therapies [15–17, 53]. Here, we developed a novel BCMA/CD47-directed UCAR-T therapy in which deletion of B2M and TRAC prevents life-threatening GvHD while effectively killing primary human MM cells and MM cell lines in vitro and in vivo. BCMA/CD47-directed UCAR-T cells demonstrated efficacy against MM and prolonged the survival of tumor-engrafted NCG mice in vivo, which provides a potential strategy for the development of a novel “off-the-shelf” cellular immunotherapies for the treatment of multiple myeloma.

CAR-T therapy has proven to be a revolutionary treatment for hematological malignancies, but the use of autologous CAR-T therapy is limited by the arguably cost, a long preparation time and inconsistent quality of autologous T cells. To overcome these problems, “off-the-shelf” CAR-T cells from allogeneic donors have been developed [54]. Up to date, there are over 60 clinical trials listed on ClinicalTrials.gov evaluating the use of allogeneic or universal CAR-T therapy in hematological malignancies. For multiple myeloma, UCAR-T cells targeting BCMA are currently being developed and have shown promising responses against multiple myeloma [55–58]. ALLO-715, a first-in-class, allogeneic anti-BCMA CAR-T therapy engineered to abrogate GvHD and minimize CAR-T rejection. Recent phase 1 clinical trial showed that MM patients (*n* = 24) treated with 320 × 10^6^ ALLO-715 CAR-T cells and a fludarabine-, cyclophosphamide- and ALLO-647-based lymphodepletion regimen achieves an ORR of 70.8%, with VGPR at 45.8% and CR or sCR at 25%, which is beneficial for patients by reducing costs and increasing accessibility [58]. Additionally, pre-clinical safety and efficacy of other allogeneic anti-BCMA CAR-T therapy against RRMM is currently being tested in a phase 1 clinical trial (P-BCMA-ALL01, NCT04960579; CB-011, NCT05722418).

The CD47-SIRPα axis, similarly to PD1/PD-L1 immune checkpoint, has emerged as a next-generation innate immune checkpoint. In recent years, several clinical trials that target CD47 have achieved excellent results [40, 59]. However, its potential adverse events (AEs) such as anemia and thrombocytopenia cannot be neglected. To address those toxicities, several anti-CD47 antibodies or SIRPα with differential characteristics have been developed. Among them are TJC4, TTI-621, ALX-148 and AO-176, which selectively bind to tumor cells while avoiding binding to erythrocytes [60–63]. TTI-621, a SIRPα-hIgG1 Fc fusion protein, selectively promotes macrophage-mediated phagocytosis by binding to CD47 on tumor cells [60]. TJC4, a differentiated fully human anti-CD47 IgG4 antibody, has unique RBC sparing properties as evident by the negligible binding to healthy human RBCs and platelets respectively. Due to the nature of the high glycosylation degree of CD47 on the RBCs, the N-linked glycan as a “shield” to block the exposure of the epitopes and prevent the TJC4 binding to human RBCs [62]. Dual-target bispecific antibodies targeting CD47 and tumor-associated antigens (TAA) or tumor-specific antigens (TSA) have been designed to alleviate hematotoxicity [64–66]. As expected, in this work, anti-CD47 antibody (CC2C6) showed strong binding and hemagglutination with RBCs, whereas the phenomena of anti-CD47 nanobodies hu404 were negligible (Fig. 2E-F); meanwhile, hu404-hFc fusion still maintain the binding ability to tumor cells (Fig. 2G). This may be attributed to a conformational change in CD47 of erythrocytes triggered by oxidative stress during aging, which causes CD47 to cluster on the erythrocyte membrane, increasing its mobility and facilitating its binding to TSP-1 [67]. The detail mechanism of anti-CD47 nanobody hu404 binds to CD47 on the tumor cells but not RBCs need further verification in the future.

CAR-engineered T cells targeting BCMA, GPRC5D, CD138, CS1 and CD38 are in active development for therapy of RRMM. BCMA-directed CAR-T cells, Abecma and Carvykti, have generated responses in MM patients, but relapses are common. GPRC5D is highly expressed in malignant bone marrow plasma cells derived from CD138 ^+^ MM patients, while its expression independently of BCMA, making it a potential excellent target for multiple myeloma therapy [68]. In a phase 1 clinical trial of GPRC5D-targeted CAR-T (MCARH109) showed that a response was reported in 71% (12/17) of the MM patients and 35% (6/17) achieved CR, 59% (10/17) achieved VGPR or better [69]. Another phase 1 trial of GPRC5D-targeted CAR-T (OriCAR-017) cells demonstrated that 100% (10/10) RRMM patients had an overall response, 60% (6/10) had a stringent complete response (sCR) and 40% (4/10) had VGPR [70]. CD138 is highly expressed on MM cells and involved in their development and/or proliferation, making CD138 an attractive therapeutic target for CAR-T cell therapy [71, 72]. In a phase 1 clinical report revealed that 80% (4/5) RRMM patients who received CD138-directed CAR-T cell therapy was in stable condition longer than 3 months and one patient had disease progression [73]. Other phase 1 trials using anti-CD138 CAR‑T are ongoing (NCT01886976, NCT03672318, NCT06006741). SLAMF7, also known as CS1, is widely expressed on the surface of plasma cells but not on nonhematologic tissues, making it a promising target for MM CAR-T therapy [74]. Several studies showed that anti-CS1 CAR-T cells exerted complete cytolysis of primary myeloma cells in vitro and in vivo and prolong mice survival [75–77]. Other phase 1 trials using anti-CS1 CAR‑T are ongoing (NCT03710421, NCT04541368, NCT04662099). CD38 is highly expressed in malignant myeloma cells, making it an attractive therapeutic target for CAR-T cell therapy. Anti-CD38 CAR-T cells are effective in eliminating primary myeloma cells as well as myeloma cell lines [78, 79]. However, CD38 is also expressed on numerous normal tissues and cells [80], which increases the potential of off-target effects of anti-CD38 CAR-T therapy in MM patients. Therefore, a rational approach for reducing on-target/off-tumor effects of anti-CD38 CAR-T cells using caspase-9-based suicide gene or affinity optimization has been developed and showed highly suitability for the generation of optimal CARs [79, 81]. Indeed, several clinical trials of anti-CD38 CAR-T cell therapy in RRMM is ongoing (NCT03464916, NCT05239689, NCT03767751). However, CD47 is also expressed on numerous normal tissues and cells, and develop a suicide gene or affinity optimization anti-CD47 CAR-T would reduce on-target/off-tumor effects.

In this work, we revealed that the cytotoxicity of BCMA/CD47-directed UCAR-T cells (h31-UCART and h32-UCART) was stronger than that of monospecific BCMA- or CD47-directed UCAR-T cells both in vitro and in vivo. The antitumor activity of h32-UCART was particularly higher compared to h31-UCART, possibly due to the use of a rigid linker (3 × EAAAK) to link hu388 and hu404. This linker facilitated the separation of functional domains by forming an α-helical structure [82, 83]. To address the issue of relapse or refractory in multiple myeloma caused by clonal heterogeneity of MM cells, Zah E et al. developed a bispecific CAR-T cells targeting BCMA and CS1, the optimized CAR-T cells exhibited robust activity against heterogeneous MM cells, outperforming T cells expressing individual BCMA or CS1-CARs [84]. Another study by Mei H et al. reported the construction of a humanized bispecific BM38-CAR that targets BCMA and CD38, which revealed that the bispecific CAR-T cells had more potent cytotoxicity against heterogeneous MM cells compared to CAR-T cells expressing only BCMA or CD38 [85].

To address the limitation of T cells lacking surface HLA class I molecules and being rejected by recipient NK cells through the “missing self” response, various strategies have been developed. Gornalusse et al. utilized gene editing to knock-in HLA-E genes at the B2M locus in human pluripotent stem cells (PSCs), disrupting HLA class I molecules and preventing NK-mediated lysis of B2M negative cells via single-chain HLA-E, thus solving a major challenge in creating universal donor cells for medical applications [86]. Wang B et al. developed a hypoimmunogenic iPSC-derived T cell that lacks B2M, MHC-II, NK cell-ligand receptor CD155, and expresses single-chain MHC class I antigen E, while maintaining anti-tumor potency [87]. These approaches aim to overcome the hurdle of NK cell activation and rejection of universal T cells. However, further exploration is still required to create “off-the-shelf” T cell immunotherapies. Interestingly, our study demonstrated that UCAR-T cells, in which TRAC and B2M genes were knocked out, did not exhibit any significant difference in cytotoxicity compared to traditional CAR-T cells (Fig. 4A, B), which suggests that TRAC and B2M gene knock-out does not alter CAR-T cell functions. Nonetheless, it is important to further investigate the potential of these strategies in the development of universal T cell therapies.

## Conclusion

In summary, we screened specific nanobodies against BCMA and CD47 protein from an immunized camel VHH library, and developed a novel allogeneic “off-the-shelf” BCMA/CD47-directed UCAR-T therapy for multiple myeloma. BCMA/CD47-directed UCAR-T cells showed potent responses against primary human MM cells and MM cell lines in vitro and in vivo, and prolonged the survival of tumor-engrafted NCG mice in vivo, which provides a potential strategy for the development of a novel “off-the-shelf” cellular immunotherapies for the treatment of multiple myeloma.

Scheme 1 Schematic presentation of VHH library construction, nanobodies screening, VHH activity detection, universal CAR-T cells preparation and anti-tumor activity analysis. (A) VHH immunized library construction against BCMA and CD47 protein. (B) Screening specific nanobodies against BCMA and CD47 protein, and detection the characterization of VHH. (C) Preparation of gene-disrupted T cells and universal CAR-T cells, and analysis the anti-tumor activity of UCAR-T cells.

### Electronic supplementary material

Below is the link to the electronic supplementary material.

Supplementary Material 1

Supplementary Material 2

Supplementary Material 3

Supplementary Material 4

Supplementary Material 5

Supplementary Material 6

## Data Availability

No datasets were generated or analysed during the current study.

## Abbreviations

MM: multiple myeloma
RRMM: relapsed or refractory multiple myeloma
PCs: plasma cells
BCMA: B-cell maturation antigen
SIRPα: signal regulatory protein-α
TSP: thrombospondin
GvHD: graft-versus-host disease
PBLs: Peripheral blood lymphocytes
HcAbs: heavy-chain only antibodies
DLBC: Diffuse Large B-cell Lymphoma
RBCs: red blood cells
PSCs: pluripotent stem cells
NK: natural killer cell
PIs: proteasome inhibitors
IMiDs: immunomodulatory drugs
Nbs: nanobodies
huNbs: humanized nanobodies
CDR: complementarity determining region
CRISPR: Clustered regularly interspaced palindromic repeats
CAR-T: chimeric antigen receptor-based T-cell
UCAR: universal chimeric antigen receptor-based T-cell
TSA: tumor-specific antigens
TAA: tumor-associated antigens
RNP: ribonucleoprotein
TCR: T cell receptor
B2M: β2-microglobulin
TRAC: T cell receptor alpha constant
sgRNAs: single-guide RNAs
HGB: hemoglobin
RTCA: Real-time cell analysis
SPR: Surface plasmon resonance
RLU: relative luminescence
iELISA: indirect ELISA
IFA: immunofluorescent assay
FCM: flow cytometry
SDS-PAGE: Sodium dodecyl sulfate-polyacrylamide gel electrophoresis
BLI: Bioluminescent imaging
TMB: tetramethylbenzidine liquid substrate
