# Supplementary material for: BCMA/CD47-directed universal CAR-T cells exhibit excellent antitumor activity in multiple myeloma
Source: J Nanobiotechnology. 2024 May 23;22:279. doi: 10.1186/s12951-024-02512-6 (PMC11112799; doi:10.1186/s12951-024-02512-6)
Supplement: Supplementary file 1 — Supplementary Material 1 [file 12951_2024_2512_MOESM1_ESM.docx]

Supplementary Table 1. gRNA sequence of TRAC, B2M and BCMA

Supplementary Table 2. TRAC, B2M and BCMA knockout sequencing data

| Gene | Mock sequencing | Knockout sequencing |
| --- | --- | --- |
| TRAC | AGACAAAACTGTGCTAGACATG  **AGG**TCTATGGACTTCAAG | AGACAAAACTGTGCTAGACATG  **AGG**TCTACTGAAGTCTCG |
| B2M | AGCGCGAGCACAGCTAAGGCCA**CGG**AGCGAGACATCTCGG | AGCGCGAGCACAGCTAAGGCCA  **CGG**AGAGATACTTCTCGG |
| BCMA | AATAACGCTGACATGTTAGAGG  **AGG**AGTATTAGAAGAACA | AATAACGCTGACATGTTAGGAAGAAGATTAAAAAAACATC |

Underline: gRNA sequence; Bold: PAM sequence.

| sgRNA | gRNA targeting sequence (5’-3’) |
| --- | --- |
| TRAC-gRNA | ACAAAACTGTGCTAGACATG |
| B2M-gRNA | CGCGAGCACAGCTAAGGCCA |
| BCMA-gRNA | TAACGCTGACATGTTAGAGG |
