# Supplementary figures and images for: BCMA/CD47-directed universal CAR-T cells exhibit excellent antitumor activity in multiple myeloma
Source: J Nanobiotechnology. 2024 May 23;22:279. doi: 10.1186/s12951-024-02512-6 (PMC11112799; doi:10.1186/s12951-024-02512-6)

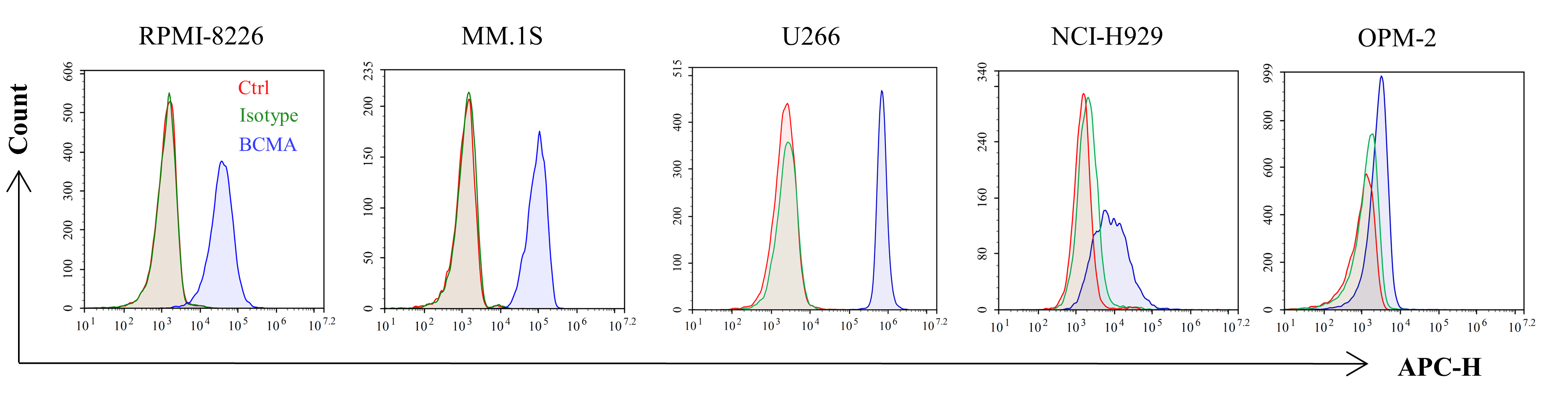

Supplement: Supplementary file 2 — Supplementary Material 2 [file 12951_2024_2512_MOESM2_ESM.tif]

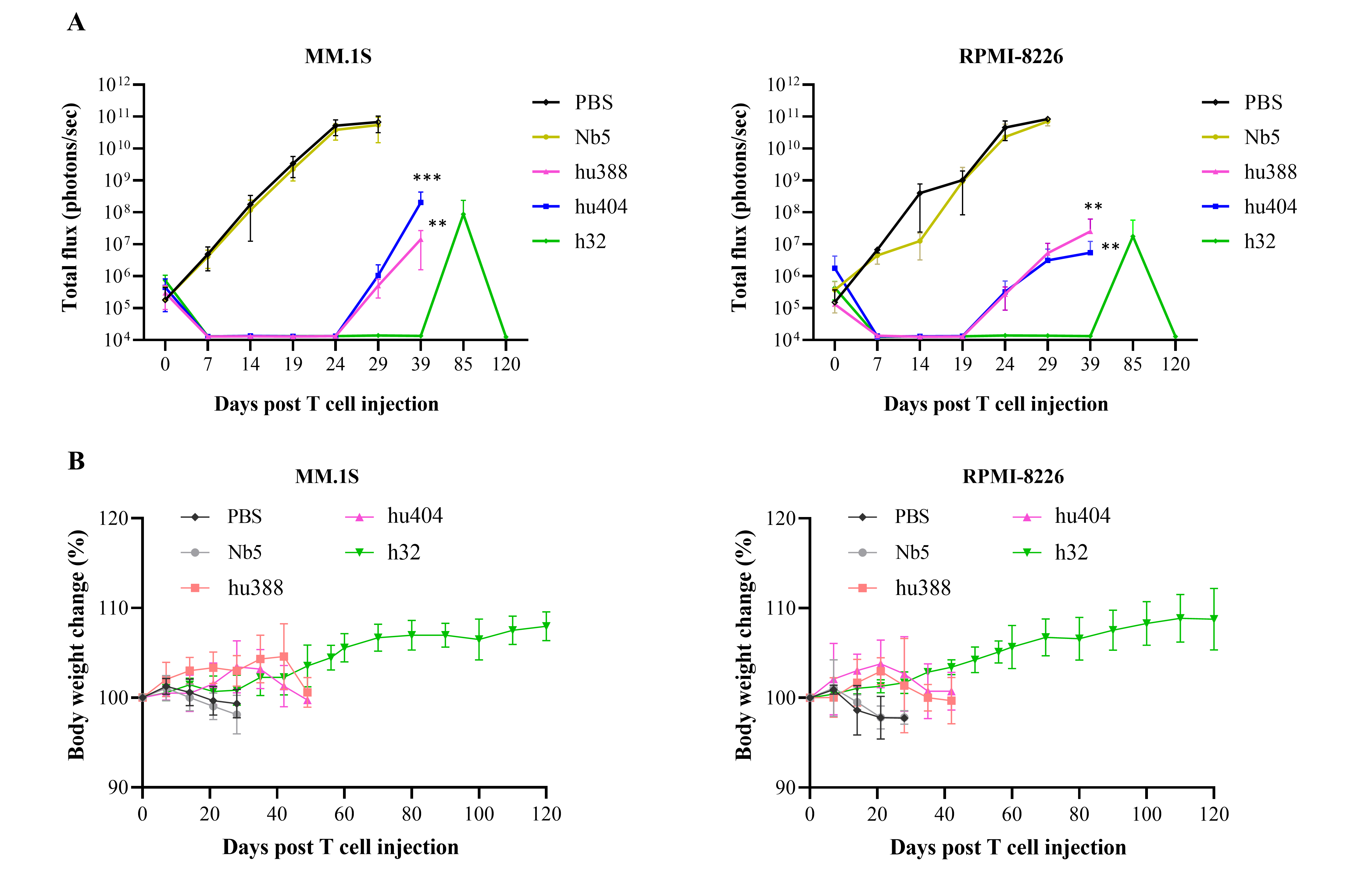

Supplement: Supplementary file 3 — Supplementary Material 3 [file 12951_2024_2512_MOESM3_ESM.tif]

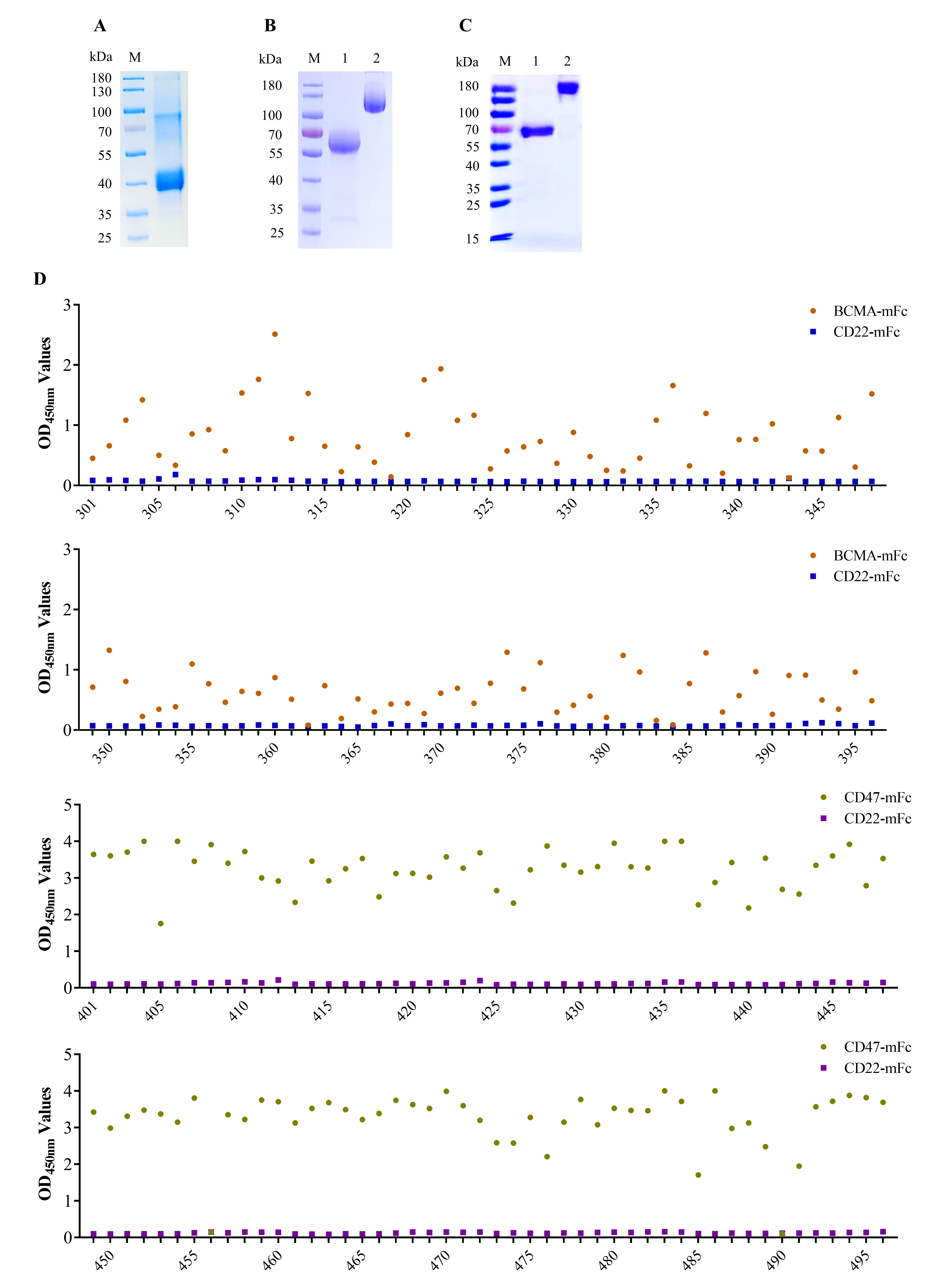

Supplement: Supplementary file 4 — Supplementary Material 4 [file 12951_2024_2512_MOESM4_ESM.tif]

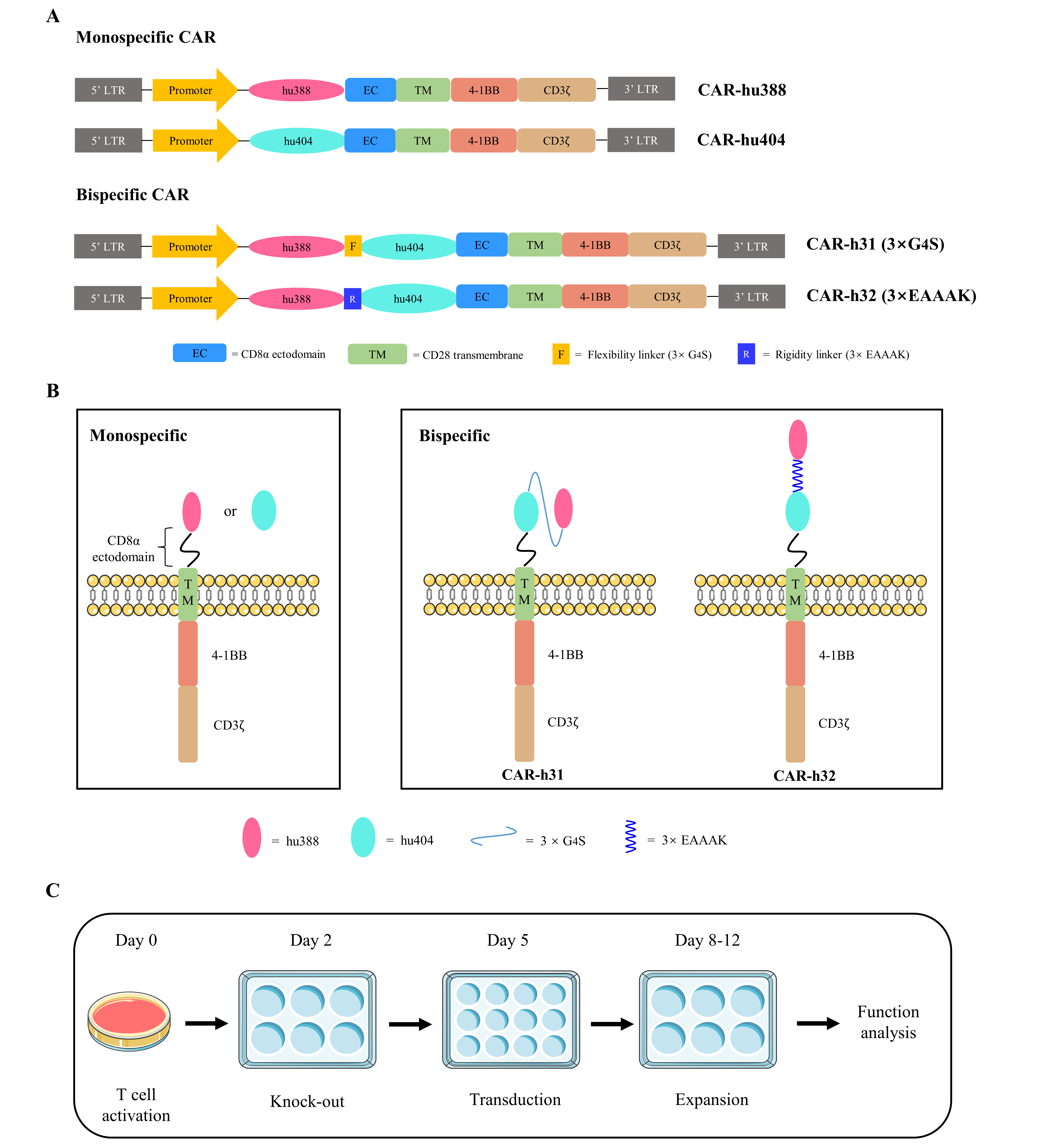

Supplement: Supplementary file 5 — Supplementary Material 5 [file 12951_2024_2512_MOESM5_ESM.tif]

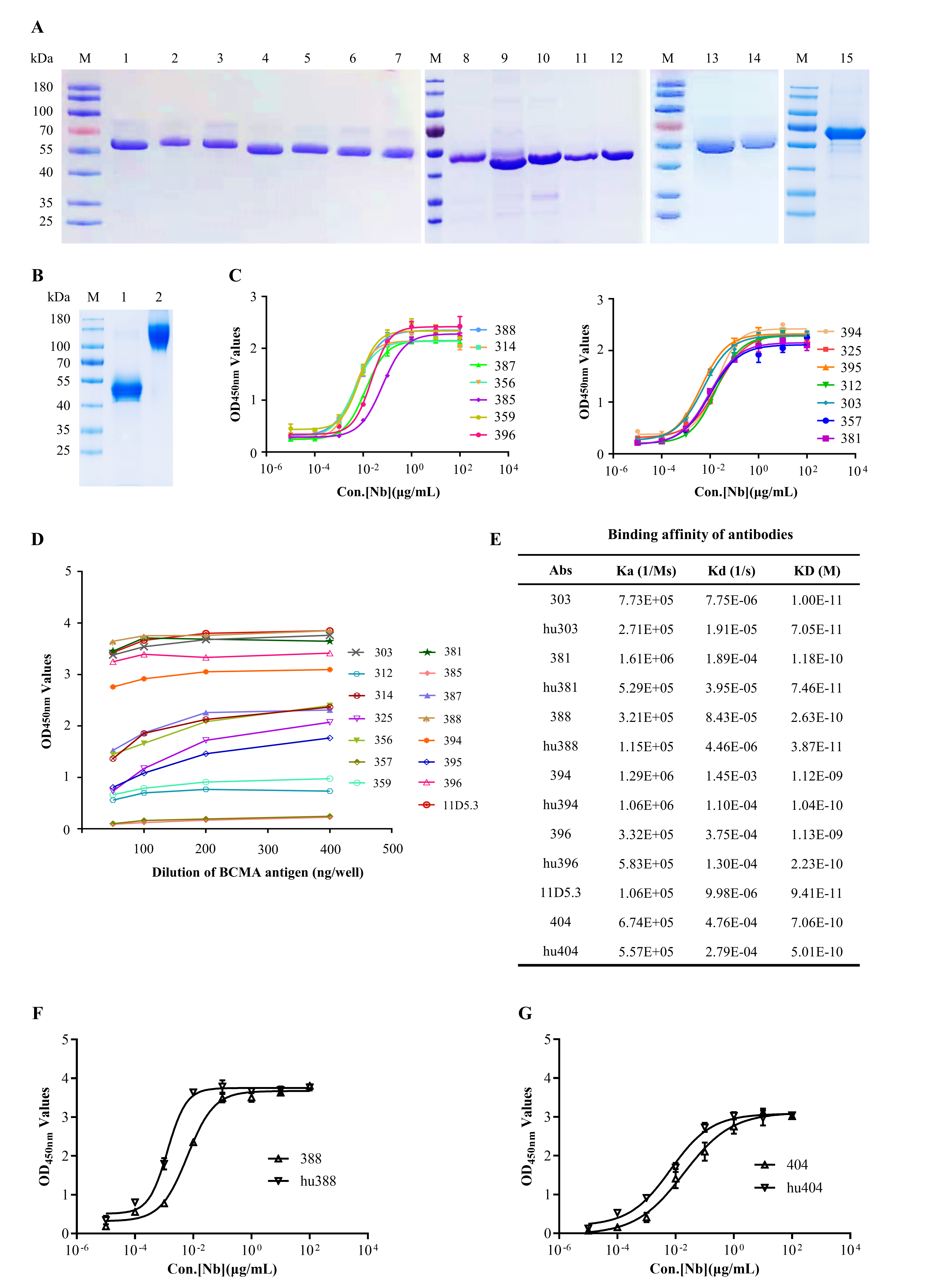

Supplement: Supplementary file 6 — Supplementary Material 6 [file 12951_2024_2512_MOESM6_ESM.tif]
